# Use of a Wireless Network of Accelerometers for Improved Measurement of Human Energy Expenditure

**DOI:** 10.3390/electronics3020205

**Published:** 2014-04-03

**Authors:** Alexander H. Montoye, Bo Dong, Subir Biswas, Karin A. Pfeiffer

**Affiliations:** 1 Department of Kinesiology, 308 W. Circle Dr., Michigan State University, East Lansing, MI 48824, USA; 2 Department of Electrical and Computer Engineering, 428 S. Shaw Ln., Michigan State University, East Lansing, MI 48824, USA

**Keywords:** artificial neural network, machine learning, ActiGraph, multi-sensor network, activity measurement, physical activity

## Abstract

Single, hip-mounted accelerometers can provide accurate measurements of energy expenditure (EE) in some settings, but are unable to accurately estimate the energy cost of many non-ambulatory activities. A multi-sensor network may be able to overcome the limitations of a single accelerometer. Thus, the purpose of our study was to compare the abilities of a wireless network of accelerometers and a hip-mounted accelerometer for the prediction of EE. Thirty adult participants engaged in 14 different sedentary, ambulatory, lifestyle and exercise activities for five minutes each while wearing a portable metabolic analyzer, a hip-mounted accelerometer (AG) and a wireless network of three accelerometers (WN) worn on the right wrist, thigh and ankle. Artificial neural networks (ANNs) were created separately for the AG and WN for the EE prediction. Pearson correlations (*r*) and the root mean square error (RMSE) were calculated to compare criterion-measured EE to predicted EE from the ANNs. Overall, correlations were higher (*r* = 0.95 *vs. r* = 0.88, *p* < 0.0001) and RMSE was lower (1.34 *vs.* 1.97 metabolic equivalents (METs), *p* < 0.0001) for the WN than the AG. In conclusion, the WN outperformed the AG for measuring EE, providing evidence that the WN can provide highly accurate estimates of EE in adults participating in a wide range of activities.

## 1. Introduction

The measurement of physical activity (PA) is beneficial for assessing the effectiveness of interventions at increasing PA, determining normal PA behaviors in populations and evaluating the associations of PA health outcomes. Use of commercial accelerometer-based activity monitors has increased in recent years, due to their utility in providing objective, valid, reliable and responsive measures of free-living PA [1,2]. Accelerometers work by collecting raw data based on accelerations of the body in one or more planes of movement, with the assumption that accelerations are proportional to muscular contraction and energy expenditure (EE). Commercially available accelerometers, such as the ActiGraph models, will digitize, filter and rectify the acceleration signals into “activity counts”, allowing meaning to be placed on the acceleration [3,4]. When accelerometers are placed on the hip, EE can be predicted from activity counts using linear regression; this method has shown moderate-to-high correlations with EE during ambulatory movement (*i.e*., walking and running), which is the most common type of movement in most adult populations [5]. However, traditional use of accelerometry suffers from several well-known limitations, including poor accuracy for the accurate estimation of the energy expenditure (EE) of activities, such as cycling, walking on an incline, climbing stairs, activities involving upper-body movement and the inability to classify the activity type [6–8]. Newer accelerometer models are able to collect raw acceleration data for days or weeks at a time at very high sampling rates; accordingly, researchers have successfully used machine learning techniques, such as decision trees and artificial neural networks (ANNs), to identify PA type, as well as intensity [9].

While machine learning algorithms developed from hip-mounted accelerometers can offer some ability to identify PA type and measure EE [7,10,11], obtaining information about movements of multiple parts of the body simultaneously can offer a greatly improved capacity for identifying PA type, intensity, frequency and duration [12]. Zhang *et al.* tested a five-sensor, wired network called the Intelligent Device for Energy Expenditure and Activity (IDEEA) and found that it was able to identify 32 types of activity with an accuracy of 98.7% [13] and could measure EE with over 95% accuracy [14]. In addition, the IDEEA monitor has been used as a criterion measure for measuring free-living EE when validating other PA measurement instruments [15,16]. However, the IDEEA has several notable shortcomings. First, the five sensors of the IDEEA are each connected (via wires) to a microcomputer worn on the hip [13], making the IDEEA cumbersome to wear. These complications significantly increase participant burden and may result in lower compliance with wearing the unit in free-living settings. Moreover, the IDEEA is not an open architecture, since it is a wired device; therefore, it cannot easily be modified to include additional sensor modalities to enhance the detection of new activities or postures. This last point is a significant limitation, since the IDEEA monitor does not have sensors mounted to the arms and is unlikely to be able to accurately detect some upper-body activities [13,17]. Finally, the IDEEA uses proprietary algorithms to classify activity type and predict EE, but the current algorithms dramatically underestimate the EE of activities, such as leg cycling [17]. In order to make a system of accelerometers suitable for free-living PA measurement, the system needs to be comfortable to wear, impose minimal burden on participants and be able to accurately measure a range of activities.

A wireless system with the ability to classify upper-body movements and to be suitable for free-living use would address many of the shortcomings of the IDEEA. Tapia *et al.* [18] were the first to use a wireless system of five accelerometers and a heart rate monitor for identifying PA type. The system achieved an accuracy of about 56% for identifying 30 activities, but was not validated or used for EE measurement, which is an important outcome variable in PA research [6]. Tapia’s system provided proof-of-concept of the utility of a wireless multi-sensor system, but their use of six measurement devices imposes a large burden on the wearer and results in a large amount of data that must be cleaned, processed and analyzed. In a later study, Intille *et al.* [19] used a three-piece wireless accelerometer network and found vastly improved EE measurement compared to hip-mounted accelerometers, but their study did not utilize ANNs, which show great promise for the improvement of EE measurement. Additionally, Intille *et al.*’s study only evaluated total EE and did not assess accuracy for measuring EE over short durations (*i.e*., minute-by-minute). Thus, over- and under-estimations throughout the course of a day could cancel each other out, yielding a misleading assessment of the system’s performance for measuring EE.

There have been several other systems of accelerometers and physiologic monitors designed for activity recognition [20–23], but there is limited research specifically on EE measurement. In a recent study, Mo *et al.* [24] were able to accurately classify activity intensity with 86% accuracy when using a system of two accelerometers and a displacement sensor (for measuring breathing rate and volume). However, activity intensity is a crude measure of EE, since there are only four intensity categories (sedentary, light, moderate and vigorous). For the purposes of determining the energy balance or movement economy, a more precise measurement of EE (metabolic equivalents (METs)) is required. Our research team recently built a three-piece wearable wireless network of accelerometers that are worn on the right wrist, thigh and ankle. The classification accuracy for 14 activities ranged from 93.4% to 97.0% in a laboratory-based setting [23], but the network has yet to be validated for EE measurement. Thus, the purpose of this study was to validate the wearable wireless network for predicting EE of 14 sedentary, ambulatory, exercise and lifestyle activities in a laboratory-based setting. A secondary purpose was to directly compare EE predictions from the wireless system to predictions from a hip-mounted, commercially available accelerometer.

## 2. Experimental Section

### 2.1. Participants

Participants in this study were 30 healthy adults aged 18–30 who were recruited from the surrounding area of East Lansing, MI. Participants were volunteers who were eligible for inclusion in the study if they were able to complete the prescribed activities and had no contraindications to exercise or gait abnormalities that would invalidate the use of accelerometry. The procedures were approved by the Michigan State University Institutional Review Board prior to the start of the study. Participants were instructed not to exercise or eat for at least three hours prior to reporting to the laboratory. Upon arriving at the laboratory, the procedures, risks and benefits of the study were described both verbally and in writing, and each participant provided written consent before beginning the study protocol. Participant height was measured using a wall stadiometer, and participant body weight was obtained using an electronic scale. Both measures were obtained according to methods described elsewhere [25]. A body fat estimate was obtained using a Quantum Labs bioelectrical impedance analysis machine (RJL systems, Clinton Township, MI, USA).

### 2.2. Equipment

#### 2.2.1. Wireless Accelerometer Network

The wearable wireless network system is comprised of three wireless MICA2 motes (Crossbow Inc., Milpitas, CA, USA). Each mote measures raw, biaxial acceleration data at a rate of 10 samples/sec (Hz) and is mounted to a battery pack and an elastic band. Together, each mote (including two AAA batteries and an elastic band) weighs about 50 grams. One mote was placed at the dorsal part of the right wrist, just proximal to the hand. The second mote was then placed on the lateral part of the right thigh, just superior to the knee, and the third mote was placed on the lateral part of the right ankle, just superior to the foot. These placements were chosen to capture the movement of the upper and lower body, as well as the postural differences between sitting, lying and standing [23]. To reduce power usage and improve the battery life of the motes, out-of-body processing was utilized, meaning that data collected by the wireless system was immediately transmitted, via a wireless signal, to a computer instead of being stored within the accelerometer (like how most commercially available accelerometers function). Accelerometer data were continuously transmitted to a computer and wireless base station. In order to minimize the loss of data during transmission, data were replicated in consecutive data packets. In the event of a data packet loss, the sensor on the base station could recover lost data due to the redundancy in the transmission. Thus, data loss would occur only when two consecutive packets were lost.

#### 2.2.2. Hip-Mounted Accelerometer

The ActiGraph accelerometer (ActiGraph, LLC, Fort Walton Beach, FL, USA) is one of the most popular accelerometers used for measurement of PA. It has been validated for use in adults and has been used in previous research using both regression equations and machine learning algorithms for estimating PA type and EE [5,7,26,27]. The ActiGraph GT3X+ was used in the current study and was set to record raw, triaxial data at a sampling rate of 30 Hz. The ActiGraph was placed on the anterior axillary line of the right hip and was secured using an elastic belt. Prior to each test, the ActiGraph was initialized using the ActiLife software according to the manufacturer’s recommendations.

#### 2.2.3. Portable Metabolic Analyzer

The Oxycon Mobile portable metabolic analyzer (CareFusion, Hoechberg, Germany) is an indirect calorimeter, which measures breath-by-breath expired gases, allowing for the calculation of oxygen consumption (VO_2_) and carbon dioxide production (VCO_2_). The Oxycon is a lightweight device (950 g) consisting of two small computer units that are mounted to participants’ backs using a shoulder harness. Participants also wear a breathing mask that covers the nose and mouth (held in place by a mesh cap) for the collection of all expired gases. The mask is attached to a digital turbine flow meter and a gas sampling tube, which connect to the computer units on the back. The flow meter and sampling tube allow for the measurement of VO_2_ and VCO_2_ from the measured inspired and expired gas concentrations and volumes. The Oxycon has been validated for the measurement of oxygen consumption across a range of exercise intensities [28], and it serves as the criterion measure of EE in this study. Before each test, the Oxycon was calibrated according to manufacturer specifications. Prior to each visit, the wireless network, ActiGraph and Oxycon were synchronized to an external clock to allow for exact comparisons of data from the devices.

### 2.3. Description of Activities Performing during the Protocol

After being fitted with the wireless network, ActiGraph and metabolic analyzer, participants began the experimental protocol. Each participant performed 14 activities in the Human Energy Research Laboratory (the activities are shown in Table 1). These activities were chosen to represent a range of sedentary, ambulatory, exercise and lifestyle activities comprising a range of intensities (from sedentary to vigorous). Activities were performed in this order, because pilot testing determined that this order corresponded to an increasing intensity of activity, thereby minimizing the time required for VO_2_ to reach a steady state for each activity [29]. The activities were performed for a duration of five minutes each. Participants were instructed to complete each activity without stopping (if possible), and between activities, they were allowed to rest and remove the Oxycon mask briefly to drink water.

For all activities, research assistants read a script to participants describing the correct execution of each activity and then were monitored throughout the visit to ensure that each activity was correctly performed. Ambulatory activities were performed on a treadmill. Cycling slow and cycling fast were performed on a Monark 818E (Monark Exercise, Varberg, Sweden) cycle ergometer, and stair climbing was performed on a StairMaster PT7000 stepmill (StairMaster Health and Fitness Products, Inc., Kirkland, WA, USA). For all sedentary activities, participants were instructed to move as little as possible. Start and stop times for each activity were recorded on a data sheet by a research assistant, and any deviations from the activity protocol or equipment malfunction were noted.

### 2.4. Data Reduction

#### 2.4.1. Artificial Neural Networks

Artificial neural networks (ANNs) are a machine learning algorithm that models the complex relationships of one or more independent variables (*x*_1_, … *x*_k_) with some outcome variable (*y*), where *k* is the number of variables used to predict *y* [9]. As their name implies, ANNs consist of a set of mathematical functions, called artificial neurons (nodes), which are interconnected. Each node calculates a summation from the input variables, and each of the inputs is assigned different weights by a weight vector. Then, activation functions are applied to each summation and combined to derive the output variable (EE). The general form of an ANN model can be seen in Equation 1, where *w* are the weights that need to be estimated, *U* is the activation function $U(z)=\frac{e^{z}}{1+e^{z}}$ (which is a linear function) and H is the number of nodes in the hidden layer.

(1) $$y=w_{0}+\sum_{i=1}^{H}\left[w_{1,i}\cdot U\;\left(w_{2,i}+\sum_{j=1}^{k}w_{j+2,i}\cdot x_{j}\right)\right]+\mathrm{Error}$$

Artificial neural networks are nonlinear regression models, but their creation is similar to the creation of linear regression models, as described elsewhere [5]. In the current study, a leave-one-out validation approach was implemented for the training and testing of the ANNs. For this approach, the training phase consisted of the measured input and output variables from all but one participant and was used to estimate the weights corresponding to each input in the ANN. Then, the ANN was tested for its ability to estimate the outcome variable from the participant left out of training. This process is an iterative process and was repeated for each participant, so that every participant’s data were used once for testing the performance of the ANN. The accuracy of the ANN was computed for each of the iterations and then averaged to obtain summary statistics about the overall validity of the ANNs.

**Figure 1:**
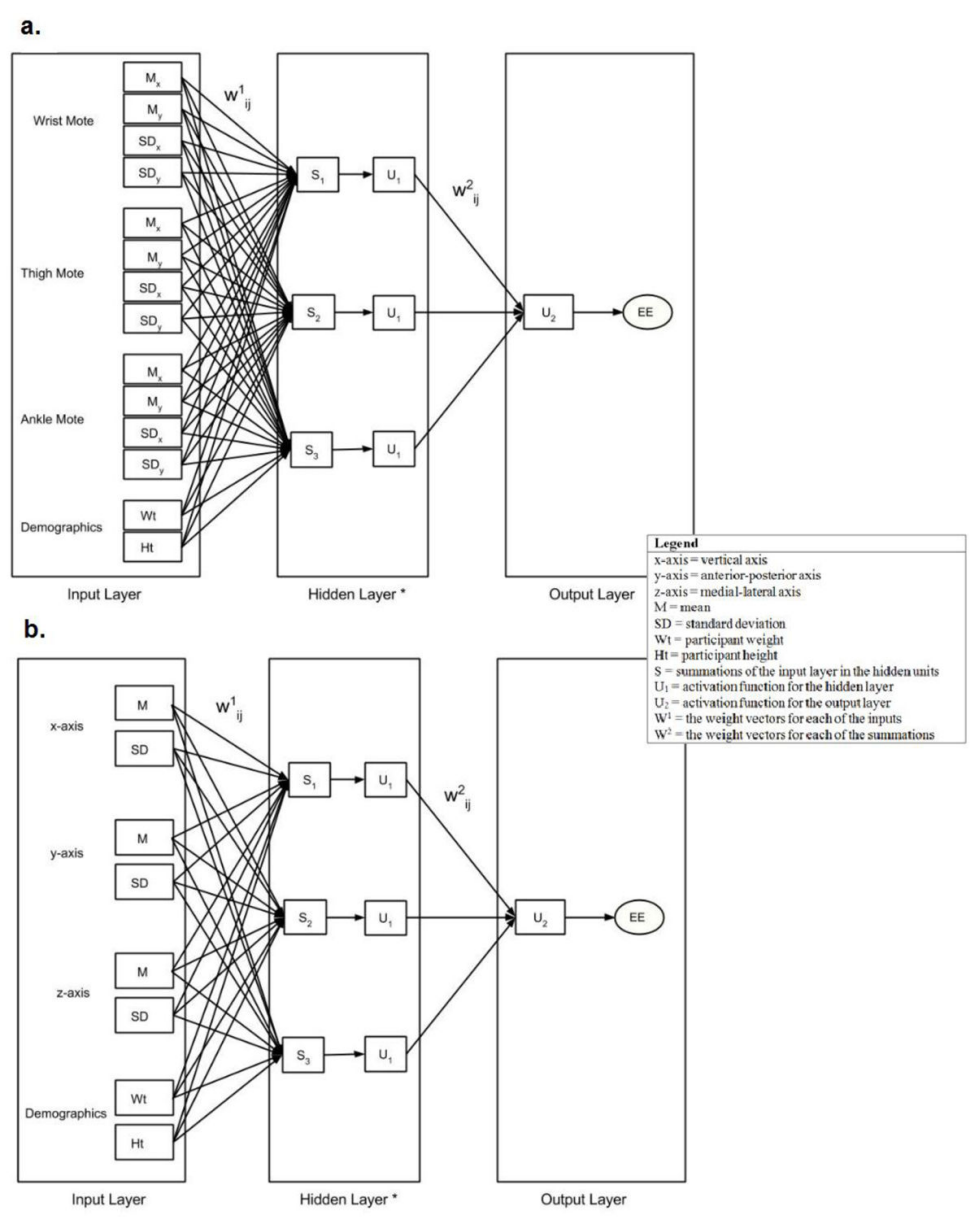
Artificial neural networks were created for predicting energy expenditure (EE) from the wireless network and the ActiGraph. (**a**) The artificial neural network (ANN) created for the wireless network; and (**b**) the ANN created for the ActiGraph accelerometer. Note that the numbers of input and output variables shown match the number used in the study, but only three hidden units are shown in the figure for simplicity. The ANNs in this study had 13 hidden units in the hidden layer.

In the current study, ANNs were created separately for the wireless network and the ActiGraph. Each ANN was created to predict EE as the outcome variable, but we used a different number of inputs to estimate EE for each ANN. In our previous work, we found that using the mean (M) and standard deviation (SD) from each axis of the collected accelerometer data as the input variables allowed for a high accuracy for classifying 14 different activities [23]; therefore, we used M and SD from each accelerometer axis as the input variables in the current study. Additionally, body size is known to affect EE; thus, participant height and weight were included as input variables. In total, the wireless network ANN had 14 total input variables (3 accelerometers × 2 axes/accelerometer × 2 inputs/axis, participant height and participant weight), and the ActiGraph ANN had eight total input variables (1 accelerometer × 3 axes/accelerometer × 2 inputs/axis, participant height and participant weight). In accordance with previous research, we included 13 nodes in the hidden layer [23]. A pictorial representation of the ANNs created in the current study can be seen in Figure 1. Calculation of the input variables and creation and testing of the ANNs was conducted using MATLAB statistical software (MathWorks, Inc., Natick, MA, USA).

#### 2.4.2. Oxycon and Accelerometer Data Collection and Processing

Oxycon-measured VO_2_ data were collected breath-by-breath and then reintegrated to 30-second windows for analysis. Relative VO_2_ was converted to METs using the following equation:

(2) $$\mathrm{METs}=\frac{VO_{2}(ml\cdot kg^{-1}\cdot min^{-1}\cdot )}{3.5}$$

To ensure that the data reflect steady-state MET values for each activity, only data from minutes 2:30–4:30 were used for each of the 14 activities.

First, M and SD from each accelerometer axis were computed in 30-second windows to allow direct comparison to EE. Raw acceleration data from the wireless system motes were collected at a sampling rate of 10 Hz, and the ActiGraph data were collected in raw mode at a sampling rate of 30 Hz. Thus, in each 30-second window, the wireless network motes recorded 300 data points, each and the ActiGraph recorded 900 data points with which to calculate M and SD for each axis of measurement.

### 2.5. Statistical Analyses

Average predicted EE values (in METs) from the wireless network and ActiGraph ANNs were calculated and compared to the measured EE for each activity. Differences between predicted and measured EE were evaluated using paired *t*-tests, and a Bonferroni correction was used to account for multiple tests being conducted.

In order to determine the validity of the wireless network for predicting EE, Pearson correlation coefficients (*r*) and root mean square error (RMSE) values were calculated for each ANN by computing the predicted EE from the ANN and comparing it to the measured EE (from the metabolic analyzer) for the data from the participant left out of the ANN creation. This was repeated for each iteration of the leave-one out validation, and the *r* and RMSE statistics computed in each iteration were averaged to obtain summary statistics of the ANN’s performance for measuring EE. The validity of the ActiGraph ANN was determined similarly. The higher the *r* values and the lower the RMSE values, the better the accuracy of the ANNs. To directly compare the validity of the wireless network to the validity of the ActiGraph, paired *t*-tests were conducted separately for RMSE and for *r* values. Since Pearson correlations are negatively skewed, we first normalized the *r* values using a Fisher’s Z transformation before performing the paired *t*-test. An alpha level of 0.05 was set for the current study; thus, *p*-values of *p* = 0.05 were used to determine significant differences in accuracy between the wireless network and the ActiGraph ANNs.

## 3. Results

The demographic characteristics of the sample are shown in Table 2. Of the original 30 participants, five participants had significant data loss from the Oxycon (due to a faulty sampling tube or battery malfunction) and two had significant data loss from the wireless network (due to a battery malfunction); therefore, only 23 were included in the final analysis. Females excluded from the final analysis weighed significantly more than females included in the analysis, causing the total sample excluded to have a significantly higher weight than those included in the final analysis. Otherwise, those included and excluded were not significantly different in terms of demographic characteristics.

**Figure 2:**
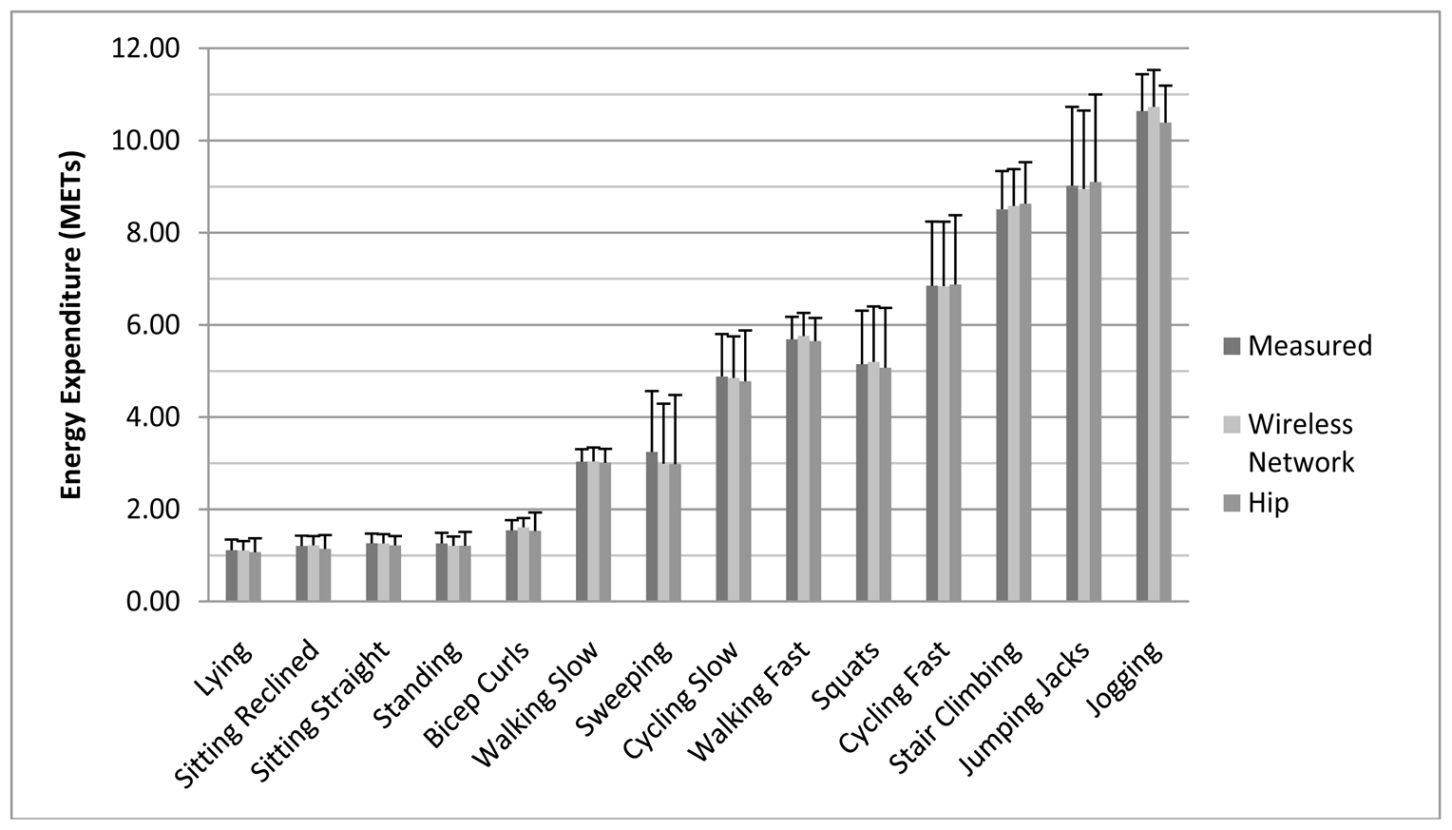
Measured EE and predicted EE from the wireless network and hip accelerometer for each activity. * Indicates significant difference from the measured EE (*p* < 0.05). METs, metabolic equivalents.

The predicted METs from the wireless network and ActiGraph ANNs compared to the measured METs can be found in Figure 2. There were no significant differences between predicted and measured METs for the wireless network or the ActiGraph ANNs for any of the activities, although the differences approached significance (*p* < 0.10) for the estimated EE from the ActiGraph ANN for sitting reclined, sitting straight and jogging, with all three activities trending toward being underpredicted compared to the measured EE. Furthermore, differences between predicted and measured EE for bicep curls approached significance (*p* < 0.10) for the wireless network ANN (trending toward overprediction).

**Figure 3:**
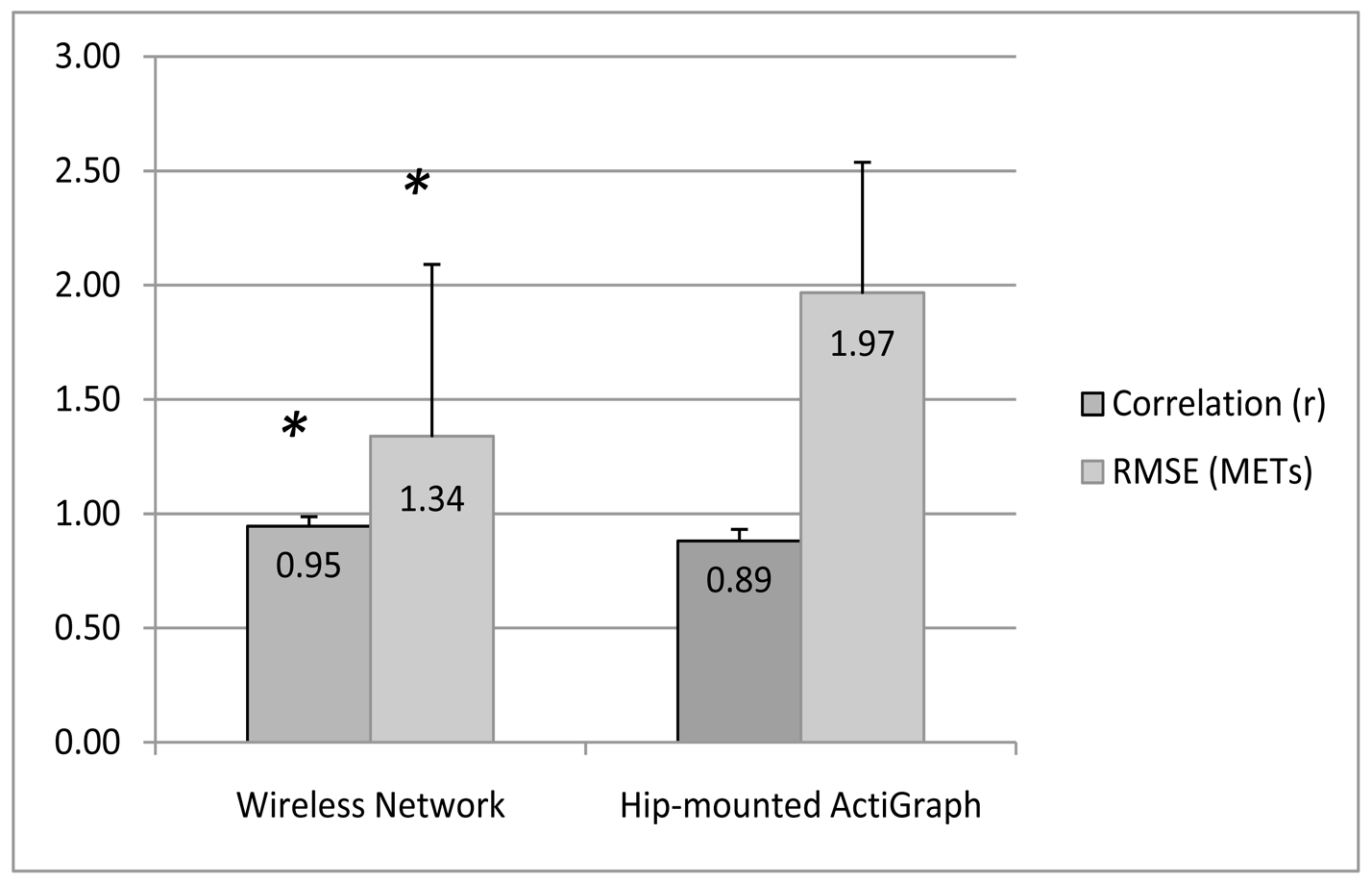
Correlation coefficients and root mean square error (RMSE) values for predicted METs from the wireless system and hip-mounted ActiGraph ANNs, compared to the measured METs. An asterisk (*) indicates significant differences from the hip-mounted ActiGraph ANN (*p* < 0.05).

Figure 3 shows the overall performance of the wireless network and the ActiGraph ANNs for predicting EE. The wireless network ANN had an average correlation of *r* = 0.95 (95% confidence interval (CI): 0.94–0.96) for predicted *vs.* measured EE. Correlations were significantly lower for the ActiGraph ANN, with an average correlation of *r* = 0.88 (95% CI: 0.87–0.90). The wireless network ANN also had a significantly lower average RMSE (1.34 METs; 95% CI: 1.04–1.64 METs) than the ActiGraph ANN (1.97 METs; 95% CI: 1.74–2.20 METs). Both ANNs showed extremely high RMSE values for Participant 23, which was surprising, given that the correlations for each ANN with this participant’s measured EE were only slightly below the average. The RMSE for Participant 23 fell well outside three standard deviations from the mean and was considered an outlier [30]. Therefore, we ran our analyses with and without Participant 23. Exclusion of Participant 23 lowered the average RMSE values for both the wireless network and ActiGraph ANNs (1.20 and 1.87 METs, respectively), but had no effect on the statistical significance of the differences seen between the wireless network and ActiGraph ANNs.

## 4. Discussion

The purpose of our study was to evaluate the performance of a wireless network of accelerometers for estimating EE using a wide range of sedentary, ambulatory, exercise and lifestyle activities. A secondary aim was to compare the accuracy of the wireless network to a hip-mounted ActiGraph accelerometer.

Overall, the wireless network ANN had high correlations for estimating EE, with an overall correlation of *r* = 0.95. This correlation between measured and predicted EE from the wireless network is similar to correlations found when predicting EE using the other monitoring systems. In one study, Zhang *et al.*, found a correlation of *r* = 0.97 between measured and estimated EE from the IDEEA monitor [14]. However, Zhang’s study used a smaller number and less diverse set of activities (11 total, eight of which were treadmill walking or jogging), which could contribute to their slightly higher correlation. In a separate study by Rothney *et al.* [11], the IDEEA monitor had a correlation of *r* = 0.91 with the measured EE in a free-living type scenario performed in a room calorimeter [11]. Together, these studies provide evidence that our wireless network can achieve similarly high accuracy for EE prediction to the IDEEA without the burden of using five sensors or a wired architecture.

Conversely, the RMSE values achieved in this study compare less favorably to the results of previous studies. Rothney *et al.*’s [11] study found an RMSE of 0.67 METs for the IDEEA, which is half of what we achieved with our wireless network. Staudenmayer *et al.* [7] achieved RMSE values of 1.22 METs for a hip-mounted accelerometer ANN when estimating EE for 18 activities, which is slightly lower than that achieved by our wireless network and much lower than the RMSE of 1.97 METs achieved with our ActiGraph ANN. It may be that our smaller sample size allowed us less data with which to train the ANNs compared to Rothney *et al.*’s and Staudenmayer *et al.*’s studies (which had 102 and 48 participants, respectively), resulting in a poorer application of the model to those not used for training. However, this seems unlikely, as an inadequate sample size would have also negatively affected our correlation coefficients. A more likely explanation of the high RMSE values, and a limitation of using RMSE for evaluating model performance when comparing among different studies, is that RMSE is not a standardized metric. Thus, a higher intensity protocol would elicit higher MET values and likely have a higher RMSE for predicting METs, simply because there is more room for error above and below the predicted value. Conversely, with protocols consisting mainly of low-intensity activities, MET values elicited by participants will stay in a relatively narrow range, resulting in smaller RMSE values. Therefore, we believe that dividing RMSE by the average EE elicited is a more appropriate metric with which to compare among studies. The average MET value achieved throughout our study protocol was 5.02 METs. This is a high MET value for a protocol lasting 70 min and is likely higher than those seen in many other studies. For example, Rothney *et al.*’s study [11] evaluated the performance of the IDEEA for estimating EE during a 24-h visit that took place in a room calorimeter. Their average caloric expenditure in their study was about 2250 kcal/day, and the average body weight in their sample was 75.7 kg, which calculates as an average of 1.18 METs over the 24-h span. Thus, their RMSE was 56.8% of their average EE, while our average RMSE was only 26.7% of the average RMSE, indicating superior performance by our wireless network compared to the IDEEA.

As previously noted, direct comparisons of the performance of the wireless network to other studies is difficult, since the number of activities and the sample characteristics will have an effect on model performance. Therefore, in the current study, we had participants wear a hip-mounted ActiGraph accelerometer in addition to the wireless network, so that we could directly compare the model accuracy. The wireless network ANN outperformed the ActiGraph ANN, yielding significantly higher correlations with EE and lower RMSE values compared to the ActiGraph. With an average correlation of *r* = 0.95, the wireless network ANN accounted for 90% of the variance observed with the measured EE, while the ActiGraph ANN accounted for only 73% of the variance in the measured EE. When comparing RMSEs between the ANNs, the wireless network ANN had an average RMSE 32% lower than the ActiGraph ANN.

Overall, both the wireless network and ActiGraph ANNs provided unbiased estimates of EE for each of the 14 activities, with predicted EE that was not significantly different than the measured EE. It is worth noting that for sitting reclined, sitting straight and jogging, there was a trend (that approached statistical significance) for the ActiGraph ANN to underpredict the EE of these activities. Previous work has shown that hip-mounted accelerometers are not as accurate in measuring sedentary behaviors as thigh accelerometers [31]; therefore, the trend toward underprediction using the ActiGraph ANN for two of the three sedentary activities (standing is not a seated or supine activity, so it is not considered sedentary [32]) indicates that placing accelerometers on parts of the body other than the hip may be preferable for measuring sedentary behavior.

Since the hip accelerometers generally have lower accuracy for measuring the EE of non-ambulatory activities [8], we expected the ActiGraph ANN to show biased EE estimates for sweeping, jumping jacks, stair climbing, bicep curls and cycling, but this was not the case. In fact, a somewhat surprising finding was the trend toward overprediction of the EE cost of bicep curls with the wireless network ANN. Since the bicep curls were performed with an unweighted broom handle, the activity involved significant arm movement, but a minimal increase in the EE cost of the activity (as is shown in Figure 2). It is likely that the increased movement detected by the wrist accelerometer in the wireless network resulted in increased EE prediction; thus, the use of a higher resistance would likely improve the EE estimates of bicep curls using the wireless network. Furthermore, while we did not observe an overall bias for the ActiGraph ANN for the non-ambulatory activities, the ActiGraph did tend to have a larger individual error in EE prediction, which is represented by the significantly higher RMSE seen with the ActiGraph ANN compared to the wireless network ANN.

Considering the superior correlations with the measured EE and the lower RMSE achieved with the wireless network ANN, our study provides strong evidence that a wireless network of accelerometers can provide improved measurement of EE compared to a single, hip-mounted accelerometer across a variety of activities. Our results are supported by a recent study conducted by Intille *et al.* [19] that found dramatically improved estimates of EE with their three-piece wireless accelerometer system compared to single-accelerometer regression equations. However, our study builds off of Intille *et al.*’s finding, because we assessed the EE over small time windows (every 30 s), whereas Intille *et al.* only evaluated the total EE. Additionally, Intille *et al.*’s study did not use ANN for their estimation of EE. Given the promising results in recent studies for classifying the activity type with ANNs, we felt that the application of these algorithms to multi-sensor systems was a vital step toward the improvement of the EE measurement. There are several limitations of the current study that should be considered. First, our sample was small and relatively homogeneous, limiting the use of our ANNs for assessing other populations with different demographic characteristics than our sample. Second, our study was conducted under highly controlled settings, where participants were instructed how to perform activities and performed each activity for the same amount of time. Additionally, we used only steady-state EE data, yet the steady state is rarely achieved in real-world settings. Finally, our included results cannot necessarily be applied to other lifestyle or exercise activities not tested in the current study. There are also several notable strengths of this study. First, our main purpose was to provide an initial validation of the wireless network for estimating EE for a variety of activities and a range of intensities. In this respect, we created our models using many common activities, as well as activities that are traditionally not measured well using hip-mounted accelerometers (*i.e*., cycling slow and fast, sweeping, bicep curls, squats, stair climbing) [33], allowing us to determine if the wireless system is able to improve the EE prediction for these types of activities. Additionally, by using a hip-mounted accelerometer and the wireless system simultaneously, we were able to draw direct comparisons between the accuracy of the two models. Finally, we used machine learning algorithms, but we also used simple-to-compute input features. The use of simple features is vital if these relatively complex data processing and analysis methods are to be adopted in a wider setting [7,9].

## 5. Conclusions

The high accuracy achieved with the wireless network for estimating EE, coupled with our previous finding that the network has high accuracy for identifying the activity type [23], indicates that our wireless network of accelerometers is a useful measurement tool that can be used for accurately assessing EE and activity classification across a range of sedentary, ambulatory, exercise and lifestyle activities. We plan to implement this network in a free-living setting to evaluate its ability to estimate free-living EE. Additionally, we plan to expand the use of this accelerometer network to measure EE in children and adolescents.

## Figures and Tables

**Table 1:** Activities and the order performed in the study. The order of activities is indicated by the number in parentheses located to the left of each activity name.

Activity Category	Activity	Description of Activity
Sedentary Activities	(1) Lying down	Participants lay still on a mat, with arms at sides and feet straight out and not crossed. Participants were not allowed to sleep.
	(2) Sitting reclined	Participants leaned back in their chair, extending their legs in front of them (while still resting them on the floor) and keeping their hands in their laps.
	(3) Sitting straight	Participants sat still in a chair with arms resting in their lap and feet flat on the floor.
Ambulatory Activities	(6) Walking slow	Participants walked at 2.0 miles/hour on a treadmill without holding handrails.
	(9) Walking fast	Participants walked at 4.0 miles/hour on a treadmill without holding handrails.
	(14) Jogging	Participants jogged at 6.0 miles/hour on a treadmill without holding handrails.
Lifestyle Activities	(4) Standing	Participants stood still, keeping feet together and arms at their sides.
	(7) Sweeping	Participants swept confetti back and forth between two cones eight feet apart. Participants swept at a self-selected pace.
	(12) Stair climbing	Participants climbed stairs on a stepmill exercise machine at a rate of 60 steps/min without holding handrails.
Exercise Activities	(5) Bicep curls	Participants performed biceps flexion and extension at a self-selected pace while holding an unweighted broom handle and standing still.
	(8) Cycling slow	Participants cycled on a cycle ergometer at 50 W (50 rpm and 1 kilopond resistance).
	(10) Squatting	Participants started with an unweighted broom handle behind the head with feet shoulder width apart. Then, participants bent at the knee until 90° flexion before returning to an upright position. Squats were performed at a self-selected pace.
	(11) Cycling fast	Participants cycled on a cycle ergometer at 75 W (75 rpm and 1 kilopond resistance).
	(13) Jumping jacks	Participants started in a standing position with feet together and hands at their sides. Then, they jumped, spreading their feet to shoulder width and extending arms upward, clapping hands together above their head before jumping back to the original position. This was performed at a self-selected pace.

**Table 2:** Demographic characteristics of the sample. (**a**) Participants included in the final analysis. (**b**) Participants excluded from final analysis.

	a. Participants Included in Final Analysis			b. Participants Excluded from Final Analysis		

	Total Sample (*n* = 23)	Females (*n* = 16)	Males (*n* = 7)	Total Sample (*n* = 7)	Females (*n* = 4)	Males (*n* = 3)
Age (years)	20.8 (1.4)	20.5 (1.4)	21.4 (1.4)	21.0 (0.8)	20.8 (0.5)	21.3 (1.2)
Height (cm)	168.5 (10.0)	163.0 (5.1)	181.1 (5.9)	173.1 (6.6)	169.3 (5.9)	178.1 (3.5)
Weight (kg)	66.0 (13.9)	58.3 (5.1)	83.4 (11.3)	77.4 (8.9) *	76.0 (11.3) *	79.3 (5.8)
BMI (kg/m^2^)	23.0 (2.6)	21.9 (1.5)	25.4 (3.0)	25.9 (3.3)	26.6 (4.4)	25.0 (1.4)
Percent fat (%)	23.9 (4.3)	26.1 (2.9)	(2.2)	21.5 (6.9)	25.9 (3.9)	15.7 (5.7)

Values are reported as the mean (standard deviation). Significant differences (*p* < 0.05) between the participants that were included and excluded are represented by an asterisk (*).
